# Visual Aura Secondary to Supratentorial Lipomatous Meningioma: A Rare Case Report

**DOI:** 10.3390/diagnostics12020365

**Published:** 2022-02-01

**Authors:** Pierfrancesco Lapolla, Placido Bruzzaniti, Giuseppa Zancana, Antonella Stoppacciaro, Michela Relucenti, Rui Chen, Xiaobo Li, Andrea Mingoli, Alessandro Frati, Pietro Familiari

**Affiliations:** 1 Nuffield Department of Surgical Sciences, University of Oxford, Oxford OX3 9DU, UK; 2 Department of Anatomical, Histological, Forensic Medicine and Orthopedic Science, Sapienza University of Rome, 00185 Rome, Italy; michela.relucenti@uniroma1.it; 3 Division of Neurosurgery, Department of Human Neurosciences, Policlinico Umberto I, Sapienza, University of Rome, 00185 Rome, Italy; placido.bruzzaniti@uniroma1.it (P.B.); giuseppa.zancana@uniroma1.it (G.Z.); pietro.familiari@uniroma1.it (P.F.); 4 Department of Clinical and Molecular Medicine, Sapienza University of Rome, 00185 Rome, Italy; antonella.stoppacciaro@uniroma1.it; 5 Department of Environmental Medicine and Toxicology, Southeast University, Nanjing 211189, China; chenrui@njit.edu.cn (R.C.); xiaobo.li@njit.edu.cn (X.L.); 6 Department of Surgery “Pietro Valdoni”, “Sapienza” University of Rome, 00161 Rome, Italy; andrea.mingoli@uniroma1.it; 7 Department of Neurosurgery, IRCCS Neuromed, 86077 Pozzilli, Italy; alessandro.frati@uniroma1.it

**Keywords:** migraine, meningioma, imaging, seizure, headache, lipomatous meningiomas, tumour

## Abstract

Background/Aim: Lipomatous meningioma is a rare type of meningioma that is formed as the result of an accumulation of lipids inside the cell due to metabolic activity dysregulation. It differs from other types of meningiomas in its radiological and immunohistochemical characteristics. We report a rare case of a patient treated in our department for this particular type of meningioma who developed a type of migraine with the aura component as the first clinical symptom. Case Report: A 55-year-old woman presented with a migraine and reported having phosphenes in recent years. Head Computed Tomography (CT) and Magnetic Resonance Imaging (MRI) scans were performed; these showed an extensive hypodense and hypointense formation located in the left parieto-occipital region. This formation was implanted in the tentorium region, with a prevailingly adipose-type signal intensity. The patient underwent an occipital craniotomy with the total removal of the lesion. The histological examination indicated a lipomatous metaplastic meningioma. Conclusion: We reported the first case of a lipomatous meningioma presenting with a migraine with a visual aura. Seizures and headaches can be included as possible symptoms. According to the current literature, lipomatous meningiomas affect women more commonly than men. The patient of our reported case presented visual disturbances in the form of a visual aura, which occurred 10 years before finding the meningioma, and surgery dramatically improved the symptoms and quality of life.

## 1. Introduction

Meningiomas constitute approximately 36.4% of all CNS neoplasms; these are benign lesions that more frequently involve the female sex. They are classified according to their histological characteristics into different subtypes; lipomatous meningiomas fall within the subtype of metaplastic meningiomas due to the accumulation of adipose tissue within the cells. They have radiological and immunohistochemical characteristics that allow for a specific diagnosis.

Headaches and epileptic seizures are the predominant clinical manifestations; this case report presents the first case of a lipomatous meningioma presenting with a migraine with a visual aura.

## 2. Case Report

A 55-year-old woman arrived at the emergency department presenting with a closed-head traumatic brain injury due to a car accident. She had a history of migraines with auras consisting of fortification spectra (expanding zigzag pattern) and transient flashing white lights, mostly recurring in the left visual field, followed by a moderate to severe left-sided throbbing headache several minutes later. 

However, the headache was not predominantly present (acephalgic migraine with visual aura). The aura episodes lasted several minutes and were associated with nausea, vomiting, photophobia, and headache, which persisted for several hours. These symptoms were relieved by sleeping and administration of oral analgesics. Analysing her medical history, the patient presented with a MIDAS score of 10 points, with grade 2, which corresponds to medium disability. During the three months before the incident, the patient experienced difficulties in carrying out daily activities and she had to take a break from work due to severe episodes of headaches. These episodes were accompanied by the visual aura that caused the patient to stay in bed and to avoid noise and light exposure. Her physical, neurological and fundoscopic examination, including blood pressure and meningeal signs, were normal. 

**Figure 1:**
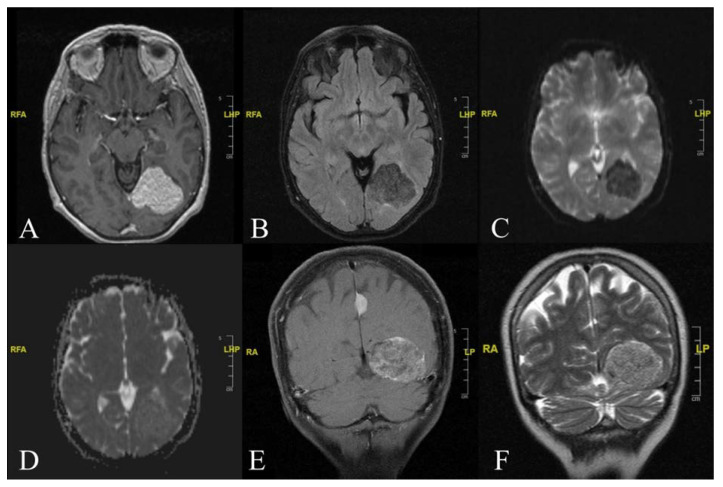
Preoperative Imaging. (**A**) Cranial T1-weighted MRI axial image with contrast showing the presence of extra-axial mass formation with of 44 × 38 mm with a craniocaudal extension of 38 mm that in the left parieto-occipital area exerts a modest compressive mass effects on the trigone and the occipital horn of the left lateral ventricle. (**B**) In the T2-weighted FLAIR sequence the lesion presents a low-intensity signal similar to fat tissue. Moderate presence of perilesional oedema indicating that the mass effect is not attributable to an oedematous process. (**C**) At Echo-Planar Two-Dimensional (EP2D) Diffusion-Weighted (DW) image, the lesion shows a low signal-intensity (low diffusion) of predominantly adipose type tissue with scarce peripheral solid component. (**D**) Similarly, the lesion displays a relative low signal intensity with relative Apparent Diffusion Coefficient (ADC); (**E**) Cranial Coronal T1-weighted MRI contrast-enhanced image showing an extra-axial mass with a relatively more inhomogeneous enhancement pattern compared to the small meningioma, 14 × 10 mm in size, arising from the cerebral falx. Comparatively, contrast; (**F**) Cranial Coronal T2-weighted MRI image reveals a high signal-intensity lesion.

Electroencephalography did not reveal any abnormalities. As part of the workup, a CT scan of the brain was obtained. Imaging showed a hypodense area of 40 × 44 mm in size within the left parieto-occipital region. Further evaluation using contrast-enhanced MRI (Figure 1) revealed the presence of an extra-axial mass arising from the left tentorial leaflet. This mass exerted compressive effects on the left lateral ventricle’s trigone and occipital horn, with no signs of cerebral oedema. 

The signal intensity of the mass showed characteristics of the adipose-tissue type with a small substantial peripheral component. MRI showed the lesion’s hyperintensity on both T1- and T2-weighted sequences, along with the contrast enhancement after an intravenous Gadolinium injection.

**Figure 2:**
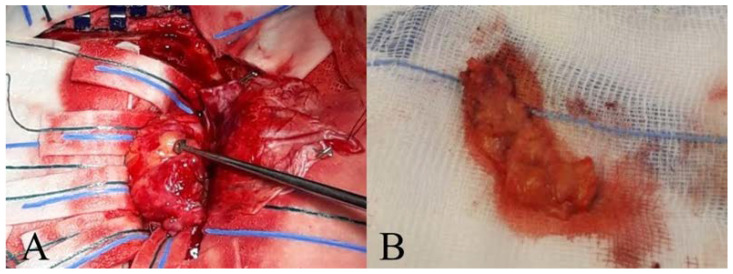
Intraoperative Photography. (**A**) A yellowish and rubbery lesion in appearance is noted. (**B**) Sample of meningioma for histological examination, after performing an “en bloc” resection.

The patient underwent a left parieto-occipital craniotomy for a Simpson grade one tumour performing an “en bloc” resection. The lesion, implanted on the superior aspect of the tentorium, appeared well-circumscribed. A clear cleavage plane between the tumour and the normal parenchyma was identified. No brain invasion was found. From intraoperative photography (Figure 2), the tumour appeared greyish (with a yellowish component) in colour and rubbery in consistency.

**Figure 3:**
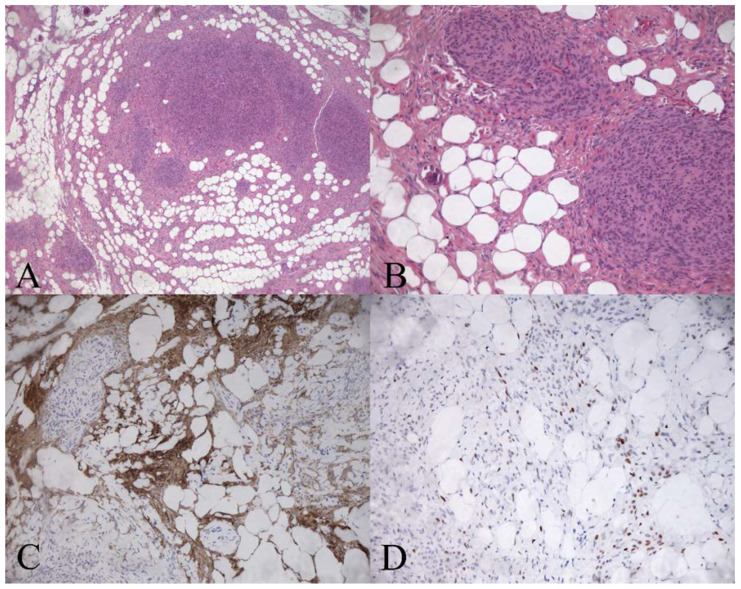
Histological examination. Metaplastic lipomatous meningioma; (**A**) Tumour tissue containing islands of typical meningothelial neoplastic cells in a field of fat-like large cells (H&E ×100). (**B**) Fat-like tumour cells have round nuclei and large fat vacuoles in the cytoplasm (H&E ×200). (**C**) Fat-like tumour cells show expression of Epithelial Membrane Antigen (EMA ×200). (**D**) Positive staining for Progesterone Receptor (PR, ×200).

The histological examination showed a mixture of meningothelial lobules and adipocyte-like cells consistent with a lipomatous meningioma diagnosis (Figure 3).

**Figure 4:**
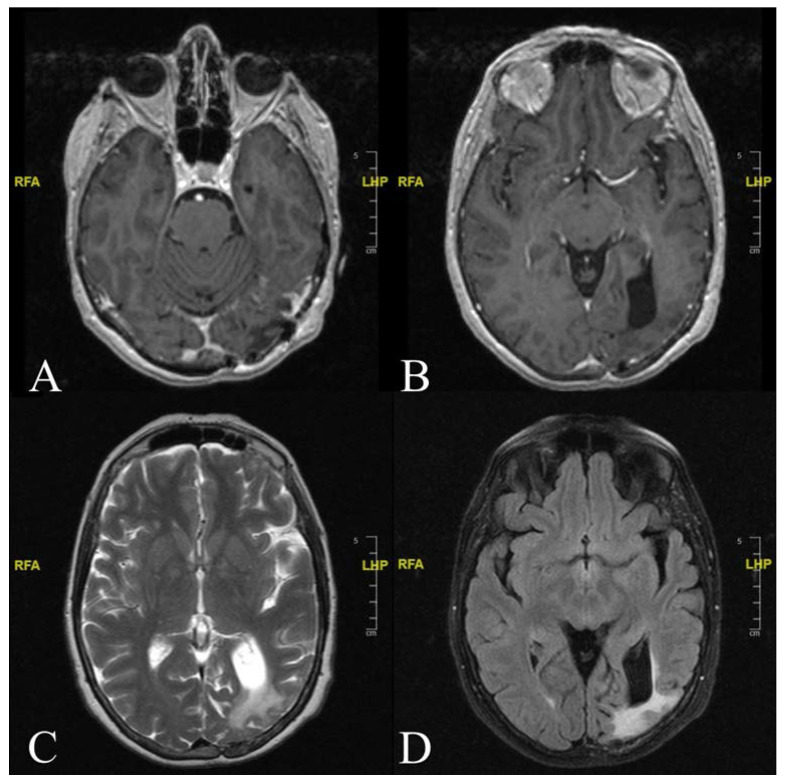
Postoperative follow-up. (**A**,**B**) Cranial Axial T1-weighted contrast-enhanced MRI image showing absence of contrast enhancement and residual disease. (**C**) T2-weighted MRI sequence revealing an asymmetry of the lateral ventricles (ALV) with ex vacuo dilation of the left occipital horn of the 4th ventricle. (**D**) Axial T2-weighted FLAIR MRI sequence showing residual parenchymal injury secondary to the compression mass effect exerted by the meningioma.

The tumour cells were positive for epithelial membrane antigen (EMA) and Progesterone Receptor (PgR). Ki-67 was <2%. The patient’s recovery and postoperative course were uneventful. MRI images at a nine-month follow-up show no sign of residual or recurrent disease (Figure 4).

She was discharged on postoperative day 7. The neurological examination at discharge was normal except for persistent transient flashing white lights. For this reason, the patient was subjected to the MIDAS-score assessment during the follow-up. The MIDAS-score result was significantly improved with respect to the pre-operative assessments. At one year follow-up, the patient was free of migraine episodes with seldom white light spots which no longer led to difficulty with daily activities.

## 3. Discussion

Lipomatous meningioma is a type of meningioma characterized by the presence of mesenchymal cells that are capable of differentiating into different subtypes, including adipose-tissue-containing cells [1]. This accumulation of fat occurs within meningothelial or transition cells [2] and is due to a metabolic cell abnormality [3].

There are extra-axial lesions with dural tails which usually appear hypodense on CT scans due to the presence of fat [4], while on MRI the appearance is variable, presenting signal loss in the sequences with fat suppression. In case the adipose-tissue component is less predominant, the area is isointense in T1 and T2 sequences [5]. The contrast enhancement appears homogeneous.

Immunohistochemically, cells of lipidized meningioma are reactive for EMA, Vimentin, CD99 and S-100 protein, and on the contrary, it tests negative for GFAP [6].

According to a recent literature review of lipomatous meningioma, there have been about 65 cases of this type [7] that had peculiar radiological and immunohistochemical characteristics typical of a lipomatous meningioma. Although a clinical examination of a lipomatous meningioma usually presents similar characteristics to common types of meningiomas, our case differs in this aspect and shows a different clinical onset of symptoms. The case reports a woman presenting with a history of migraines with visual-aura components; the patient describes these episodes as flashes of white light followed by persistent headaches, nausea, vomiting and photophobia. Additionally, the patient reported that these symptoms had a negative impact on daily life and had been compromising daily activities for years. The finding of the meningioma was accidental and was found when the patient underwent a CT scan for a head injury. The finding of this formation was treated with a surgical approach, performing a total removal of the meningioma followed by a histological examination of the tissue. The resection of the mass was a total removal of the formation. The patient had no relapses and after one year she reported a complete remission of all the related symptoms.

Headaches can be considered one of the most frequent clinical manifestations of the presence of intracranial masses. Interestingly, an imaging-analysis investigation is more commonly performed for patients presenting with a new onset of a headaches than those with chronic headaches. As for patients with a history of chronic headaches, Whang et al. reported that about 3.7% of these patients have abnormal radiological findings [8]. In addition, Schankin et al. carried out a study on 58 patients with meningiomas and about 40% of cases presented with headaches; only 22% of these were migraines [9]. However, the correlation between meningiomas and migraines with auras is quite rare and the cases reported in the literature are described as a type of secretory meningioma [10] and a meningothelial meningioma [11]. It has been shown that intraoperative stimulation of exposed dura-mater sites cause an increase in pain [12], especially in the affected areas of the trigeminus. However, in addition to direct stimulation of the dural afferents of both vascular and nervous origin, there is a direct correlation between the size of the tumour and the diameter of the surrounding oedema, probably related to the dislocation of the painful structures [13]. Additionally, a correlation was seen between pronociceptive chemicals and the presence of headaches; high concentrations of PGE2 and substance P could promote the activation of a pro-nociceptive cascade with neurotonic inflammation [13].

Our reported case is the first case of a lipomatous meningioma presenting with a history of migraines with visual auras. Based on these findings, various hypotheses arise that can account for the observation regarding the cause of the migraine: there may be traction on vascular structures, compression on the cranial nerves, a neurogenic inflammation through vascular afferents to the meninges or on the cranial vessels [14]; however, the specific causes are not yet defined.

## 4. Conclusions

Lipomatous meningioma differs from other meningiomas due to its peculiar clinical, radiological and immunohistochemical characteristics. Seizures and headaches can be the primary onsets of symptoms in lipomatous meningioma. Additionally, precise immunohistochemical findings should be correlated with imaging features that help in the earlier identification of the tumour. A defined diagnosis of lipomatous meningioma is a crucial factor in the choice of surgical intervention. In conclusion, from our case reported here, we observed that the complete resection of the meningioma (Simpson grade one removal) led to the resolution of migraines with auras, and after one year the patient presented with a significant improvement in daily life activities.
